# Pipelle endometrial biopsy for abnormal uterine bleeding: do patient’s pain and anxiety really impact on sampling success rate?

**DOI:** 10.1186/s12905-021-01526-8

**Published:** 2021-11-12

**Authors:** Aiym Kaiyrlykyzy, Faina Linkov, Faye Foster, Gauri Bapayeva, Talshyn Ukybassova, Gulzhanat Aimagambetova, Kamila Kenbayeva, Bakytkali Ibrayimov, Alla Lyasova, Milan Terzic

**Affiliations:** 1 National Laboratory of Astana, Nazarbayev University, Kabanbay Batyr Street, 53, 010000 Nur-Sultan, Kazakhstan; 2 Department of Health Administration and Public Health, John G. Rangos, Sr. School of Health Sciences Duquesne University, 600 Grant St, Pittsburgh, PA 15282 USA; 3 Department of Obstetrics, Gynecology and Reproductive Sciences, University of Pittsburgh School of Medicine, 300 Halket St, Pittsburgh, PA 15213 USA; 4 Department of Medicine, Nazarbayev University School of Medicine, Zhanybek-Kerey Khans Street, 5/1, 010000 Nur-Sultan, Kazakhstan; 5 Clinical Academic Department of Women’s Health, National Research Center of Mother and Child Health, University Medical Center, Turan Ave. 32, 010000 Nur-Sultan, Kazakhstan; 6 Department of Biomedical Sciences, School of Medicine, Nazarbayev University, Zhanybek-Kerey Khans Street, 5/1, 010000 Nur-Sultan, Kazakhstan; 7 Clinical Academic Department of Laboratory Medicine, Pathology and Genetics, University Medical Center, Republican Diagnostic Center, 2 Syganaq Street, Nur-Sultan, 010000 Kazakhstan; 8 Pathology Bureau of Nur-Sultan City Administration, Zhansugirov Street, 1, 010000 Nur-Sultan, Kazakhstan

**Keywords:** Pipelle endometrial biopsy, Pipelle sampling, Endometrial sampling, Pipelle failure, Pain, Anxiety

## Abstract

**Background:**

Pipelle endometrial biopsy is vital for the early diagnostics of endometrial pathology and is performed in outpatient setting in minimally invasive manner. One of the reported disadvantages of sampling with Pipelle curette is failure to collect enough tissue for histological analysis. The role of psychological factors such as anxiety and pain sensitivity in obtaining adequate samples is not well known. The study's objective was to explore whether there is relationship between severe pain, anxiety, and the rate of Pipelle failure.

**Methods:**

Study included 158 women with median age of 42 who underwent Pipelle endometrial biopsy at Clinical Academic Department of Women’s Health of the University Medical Center (UMC), Nur-Sultan City, Kazakhstan with an abnormal uterine bleeding from June 2019 to April 2021. Women were asked to fill survey on pain, anxiety before, during and after the procedure.

**Results:**

3.8%, 15.19% and 4.43% of women reported severe pain and 39.24%, 34.18% and 14.56% of women reported severe anxiety prior, during and after procedure, respectively. Women who experienced severe pain during procedure tend to be more anxious during procedure (*p* = 0.0001) and have higher number of sampling attempts (*p* = 0.0040). Pain level was higher among patients sampled by the junior OB/GYN specialist (*p* < 0.0001). We found no differences in Pipelle biopsy success rates in relationship to baseline, during and postprocedural pain and anxiety scores.

**Conclusion:**

Anxiety during procedure performance was significantly associated with severe pain during the procedure but did not represent a key element for the success of Pipelle biopsy.

## Introduction

Endometrial cancer (EC) is the most common gynecologic malignancy in the developed world. Due to increasing obesity rates and little access to preventive services, EC mortality in Kazakhstan is higher than in comparison to the US and other developed countries. Office vacuum aspiration (Pipelle) for endometrial biopsy (EMB) sampling plays an important role in early cancer diagnosis, preoperative assessment, and treatment planning for endometrial pathologies [1]. Use of a Pipelle suction curette (flexible plastic curette used for minimally invasive tissue collection) has emerged as the most common method for endometrial tissue sampling [2], often indicated for evaluation of abnormal uterine bleeding [3, 4], which is responsible for ~ 70% of all peri- and post-menopausal gynecological visits [5]. EMB has been reported to be an equivalent and sometimes even superior tool for detecting EC compared to other endometrial sampling techniques [2] with diagnostic accuracy comparable to dilatation and curettage (D&C) sampling for some types of endometrial pathology, including endometrial hyperplasia, hyperplasia with atypia, and EC [3, 6, 7]. Compared to D&C sampling conducted in the operation room, EMB is relatively inexpensive, associated with less morbidity, safe, accurate, and can be performed in an office setting [2].

Despite the documented benefits there are risks for failure associated with Pipelle procedure, one of the main being the failure to obtain satisfactory samples for histological examination [2]. Thus, it is important to establish factors that may have an impact the ability to collect an adequate endometrial sample.

Many invasive diagnostic procedures have been reported in the literature to induce discomfort in patients by causing anxiety [8, 9]. This is supported by a substantial body of evidence that links elevated state anxiety to an increase in pain intensity and decrease in pain tolerance [10, 11]. For example, level of anxiety affects the tolerability of the office hysteroscopy [9, 12]. The role of psychological factors such as anxiety and pain sensitivity in obtaining adequate samples, have rarely been investigated as an increased risk of biopsy failure.

The Pipelle procedure has shown better or equal tolerability by patients compared to other office-based devices [13, 14], however, although most women tolerate it well, some do experience significant discomfort, which may affect the efficacy of the procedure. Adambekov et al. suggest the strong links between anxiety before a Pipelle biopsy and patients' experiences of extreme pain during the procedure [1, 15].

This study has been designed to improve our understanding of acceptability and factors influencing successful use of EMB by investigating the relationship between severe pain, anxiety, and the rate of Pipelle failure.

Taking into account that Kazakhstan has a higher rate of mortality due to EC than in western countries, and despite EMB reported to be a preferred modality for diagnosing gynecological pathologies in the Western world, EMB is not widely utilized in the clinical practice as a diagnostic tool in Kazakhstan. The main method of endometrial sampling in Kazakhstan remains D&C, which requires hospitalization, anesthesia, antibiotic use, and is more invasive. By improving our understanding of acceptability and factors influencing successful use of EMB, the results of this study will take us one step closer to enabling the timely diagnosis of current endometrial pathology, will have an important impact on healthcare safety and efficiency, and improve overall treatment outcomes and the quality of life of Kazakhstani women.

## Methods

We performed an observational study on data of women who met the criteria for endometrial biopsy. These criteria/indications included abnormal uterine bleeding, pre- and postmenopausal bleeding. Recruitment took place from June 2019 to April 2021 at the Clinical Academic Department of Women’s Health of the University Medical Center (UMC), Nur-Sultan City, Kazakhstan.

Inclusion criteria included: female; age 18+; with an intact uterus and cervix; endometrial biopsy recommendation due to (but not limited to) abnormal uterine bleeding and irregular cycles (for pre-menopausal women) or post-menopausal problem bleeding.

Exclusion criteria included: cervical cancer, pregnancy, acute pelvic inflammatory disease, clotting disorders, acute cervical or vaginal infection, uterine anomalies/malformations, hysterectomy, previous endometrial ablation, or any intervention/procedure done for Asherman.

Endometrial sample collection: The Pipelle endometrial sampling was carried out in the gynecological outpatient clinic of the UMC. If the tissue obtained was considered inadequate under visual assessment, the procedure was repeated to optimize sampling. The endometrial tissues obtained were fixed in 10% buffered formalin and transported to the pathology laboratory for histopathological studies. The patient was then transferred to the operating room for D&C under general anesthesia. The D&C was performed according to hospital protocols and the endometrial tissues were fixed in 10% buffered formalin as described above. Procedures were performed by one senior (> 35 years of experience) and one junior (< 5 years of experience) specialists in obstetrics and gynecology (OBGYN). Required training for OBGYN specialists in Kazakhstan is minimum of three-year residency program. The coupled endometrial biopsy samples were subjected to histopathological studies. Histopathological evaluation and diagnosis included all morphologic abnormalities that were observed in the coupled samples.

Biopsy failure was defined as the inability to access the uterine cavity or the inability to obtain a sufficient amount of tissue for histological examination. Two independent experts in gynecologic histopathology evaluated the obtained specimens and followed the guidelines for classification of entities related to endometrial pathological findings.

Pain intensity before, during and after Pipelle sampling was evaluated using Numerical Rating Scale (NRS) [16], where “0” represents no pain at all, “10”—the worst pain ever possible. Pain rating was categorized as 1–6—mild to moderate, 7 and above as severe pain. Anxiety was assessed using 0–10 numeric rating, where “0” represents no anxiety, “10”—As anxious as I could be. Demographic and clinical data were obtained from each participant during a medical interview.

Descriptive statistics were reported as median (IQR) for continuous variables and n (%) for categorical variables. Comparison of patients’ characteristics by pain intensity groups (no pain, mild to moderate pain and severe pain) was performed using Kruskal–Wallis test. A binomial logistic regression was run to assess the relationship between anxiety and pain. Comparison of anxiety and pain scores between biopsy outcome groups was performed using non-parametric Mann–Whitney U test. *P*-values < 0.05 were considered significant. All statistical analyses were performed with Stata 16 (StatCorp).

## Results

**Fig. 1:**
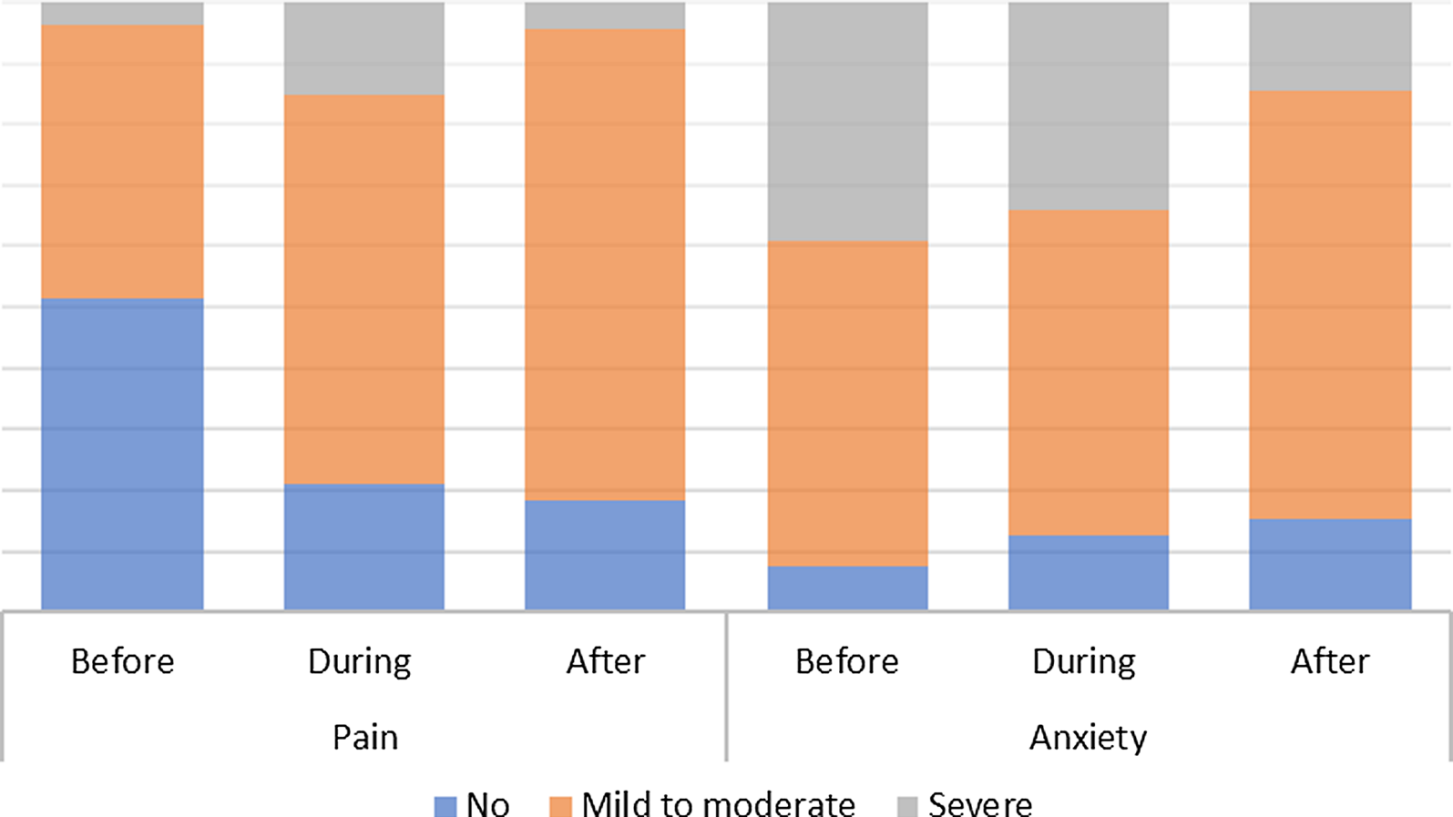
Pain and anxiety before, during and after procedure

The study included 158 patients, with a median age of 42 (34–48.3) years old, with 30 (18.99%) women who were postmenopausal. Inadequate biopsy samples were obtained in 25 out of 158 patients (18.8%). Severe pain prior, during and after procedure experienced 3.8%, 15.19% and 4.43% of women, respectively. 39.24%, 34.18% and 14.56% of women reported severe anxiety before, during and after Pipelle sampling, respectively (Fig. 1).

The median pain score during procedure was 2 (0–4) with the senior OB/GYN specialist, while women who underwent Pipelle sampling with a junior specialist reported median biopsy pain as 5 (4–7) (*p* < 0.0001). No significant relationships were observed between pain experienced and menopausal status, provider and biopsy outcome.

In Table 1 we outlined patients’ age, BMI, anxiety prior and during procedure, number of attempts to obtain sample by reported pain intensity. Women who experienced severe pain during procedure tend to be more anxious during procedure (*p* = 0.0001) and have higher number of sampling attempts (*p* = 0.0040) compared to those who had no or mild to moderate pain.

**Table 1:** Comparison of patients by pain intensity groups

	Pain intensity during procedure (0–10)			*P *value*
	No pain (0) N = 33 (20.89%)	Mild to moderate (1 to 6) N = 101 (63.92%)	Severe pain (7 and above) N = 24 (15.19%)	
Age	35 (31–45)	43 (35–49)	42.5 (35–46.5)	0.0713
Body Mass Index	26.57 (21.48–30.38)	25.6 (22.39–29.74)	26.49 (23.7–30.63)	0.6803
Anxiety before procedure	5 (1–7)	6 (3–8)	5.5 (4–7)	0.1419
Anxiety during procedure	1 (0–4)	5 (2–7)	8 (7–8)	0.0001
Anxiety after procedure	2 (0–4)	3 (1–5)	5.5 (4–7)	0.0001
Number of biopsy attempts	1 (1–3)	2 (1–3)	3 (2–3)	0.0040

*Kruskal–Wallis test was performed to compare differences in patients’ characteristics between pain intensity groups

A binomial logistic regression was used to predict pain based on anxiety. Results show that anxiety during procedure performance was significantly associated with severe pain during the procedure (OR = 1.66 (95% CI 1.32, 2.07), *p* < 0.001). After adjusting for anxiety before procedure, number of biopsy attempts, type of provider and women menopausal the relationship remained significant (OR = 1.85 (95% CI 1.24, 2.55, *p* < 0.001).

In our study results we did not observe any significant association between pain and the Pipelle biopsy outcome.

## Discussion

In our study, anxiety during procedure performance was significantly associated with severe pain during the procedure but did not represent a key element for the success of Pipelle biopsy.

One of the reported issues with Pipelle sampling is the inability to obtain sufficient samples for histological analysis. However, the role of patient’s psychological factors such as anxiety and pain perception in obtaining adequate samples is not clearly understood. Thus, the objective of this study was to evaluate the relationship between severe pain, anxiety, and Pipelle failure rate.

Even though Pipelle biopsy is regarded as a painless or mildly painful procedure when compared to other approaches, we found that nearly half of the patients experienced pain of varying degrees during the procedure, and that pain was increased with providers of less experience*.* Moreover, we observed anxiety during procedure was associated with severity of pain during procedure, which is in line with Adambekov et al.’s [15] finding that extremely anxious patients before the procedure have 2.3 times higher chances of severe pain during the procedure.

Participant’s pain and anxiety were more intense in the process of sampling, which is an expected result since Pipelle endometrial sampling usually performed without use of anesthesia [17]. Consistent with previous studies, pain intensity during endometrial sampling depended on the experience of health care provider, number of Pipelle passes made and patient’s anxiety during procedure [1, 15, 18].

One of the most important factors associated with office hysteroscopy failure has been reported to be pain [12, 19], our study however found that neither pain nor anxiety scores affected Pipelle biopsy success. Furthermore, although it has been reported that Pipelle is more painful, thus less acceptable for postmenopausal aged women [20–22], we did not find significant difference in pain scores between pre and postmenopausal women.

Although anxiety did not represent a key element for the success of Pipelle biopsy, our findings suggest the patient experience of endometrial biopsy procedure could be considerably improved by measures aimed at reducing anxiety. A clear description provided before the procedure, as well as psychological support during the procedure, may improve the patient's acceptance of the predictable pain associated with an endometrial biopsy. This is an important finding considering that in the outpatient environment, the ideal endometrial biopsy approach is simple to operate and obtains a sufficient endometrial sample without causing discomfort to the patient. It is important that health care providers ensure that patients are relaxed and that procedures are conducted safely as minor gynecologic procedures transfer from the operating room to the office [23].

Kazakhstan is one of the post-soviet republics of Central Asia. Collapse of the Soviet Union led Kazakhstan to economic recession and the healthcare systems of the country has gone through decades of profound revolutions. Recently Kazakhstan moved from lower middle-income to upper middle-income country according to the World Bank classification. However, despite recent improvements in health care in response to the reforms, Kazakhstan still lags behind other post-soviet independent states with only 3.4% of GDP allocated for healthcare system.

For the past years, EMB in Kazakhstani gynecological practice was done mostly using classic D&C procedure due to lack of access to the Pipelle tool as a new technique requiring additional budget. Moreover, Pipelle biopsy utilization guideline was approved by the Ministry of Healthcare of the Republic of Kazakhstan only in 2018. Now, Pipelle is among the suggested/approved methods for endometrial biopsy and is increasingly used for monitoring of endometrial histology during hormone therapy. Although it is in use now, but only in the large cities’ tertiary care hospitals, which can afford to buy the Pipelle sampling tool.

Introduction of EMB in Kazakhstan ambulatory care settings is needed to improve the rate of early diagnosis of endometrial pathologies and improve overall patient outcomes. For patients who have indications for D&C, introducing Pipelle biopsy in the office settings would be a safe, reliable, and cost-effective outpatient procedure for diagnosing endometrial pathologies, including endometrial cancer.

Nonetheless, the study has some limitations. Since group stratification by biopsy result is based on small sample of women, it should be considered exploratory. Secondly, we did not utilize the Spielberger State-Trait Anxiety Index (STAI) and thus did not differentiate a present anxiety state from a long-term trait anxiety. This study will however, serve as the groundwork for future research aimed at the development and testing of new intervention strategies for increasing the use of EMB rather than D&C sampling for endometrial cancer diagnoses in Kazakhstan.

## Conclusion

The overall goal of this study was to fill important gaps in the current understanding of factors relevant to attempting and conducting successful EMB Pipelle endometrial biopsy and guide clinicians to choose an appropriate approach for obtaining an endometrial sample in a particular patient and current circumstances.

We found that anxiety and pain did not impact on Pipelle success rate, but that OBGYN specialists experience and anxiety maybe considered predictors of procedural pain, highlighting the need for health care providers, particularly less experienced providers, to ensure that patients are relaxed to guarantee that procedures are conducted safely as minor gynecologic procedures.

In the future, this study could have a significant impact at both individual patient and healthcare level in Kazakhstan. Proper patient information/education, understanding of Pipelle tool sampling benefits, including lower pain and anxiety, would facilitate a wide implementation of the technique across the gynecological settings in the country, especially in the private clinics. We hope this study will have a positive impact on strategies for providing high-quality care for patients.

## Data Availability

The datasets used and/or analysed during the current study are available from the corresponding author on reasonable request.
